# Introductory Lecture on the Theory and Practice of Medicine in the Pennsylvania College

**Published:** 1847-03

**Authors:** W. Darrach


*Introductory Lecture on the Theory and Practice of Medicine in the Pennsylvania College,* by W. Darrach, M. D.

The lecturer chose for his subject—"Life;" and truly he shows himself fully competent to discuss this mooted and vexed subject. We regret that we have not room for extracts, but suffice it to say, that it is valuable as a literary, as well as a physiological production.

